# KINLI: Time Series Forecasting for Monitoring Poultry Health in Complex Pen Environments

**DOI:** 10.3390/ani15213180

**Published:** 2025-10-31

**Authors:** Christopher Ingo Pack, Tim Zeiser, Christian Beecks, Theo Lutz

**Affiliations:** 1 Data Science & Artificial Intelligence, Fraunhofer Institute for Applied Information Technology FIT, 53757 Sankt Augustin, Germany; christian.beecks@fit.fraunhofer.de; 2 Digital Supply Chain Research Group, Offenburg University of Applied Science, 77652 Offenburg, Germany; theo.lutz@hs-offenburg.de; 3 Institute for Machine Learning and Analytics, Offenburg University of Applied Science, 58084 Offenburg, Germany; 4 Fakultät für Mathematik und Informatik, Fern Universität in Hagen, 77652 Hagen, Germany

**Keywords:** large language models, transformers, deep learning, time series, time series forecasting, animal farming

## Abstract

We analyze how to perform accurate time series forecasting for monitoring poultry health in a complex pen environment. To this end, we make use of a novel dataset consisting of a collection of real-world sensor data in the housing of turkeys. The dataset comprises features such as food intake, water intake, and various environmental values, which come with high variance, sensor defects, and unreliable timestamps. In this paper, we investigate different state-of-the-art forecasting algorithms to predict different features, as well as a variety of deep learning models such as different transformer models and time series foundational models. We evaluate both their forecasting accuracy as well as the efforts required to run the models in the first place. Our findings show that some of these aforementioned algorithms are able to produce satisfactory forecasting results on this highly challenging dataset while still remaining easy to use, which is key in a tech-distant industry such as poultry farming.

## Simple Summary

The paper presents the KINLI project, which applies machine learning and deep learning techniques to time series forecasting for monitoring turkey health in poultry farms. Using a real-world dataset from turkey barns—characterized by noisy, incomplete, and irregular sensor data (e.g., food intake, water intake, environmental factors)—the study evaluates a wide range of forecasting models. These include statistical approaches (ARIMA and Prophet), classical machine learning models (XGBoost and LSTM), transformer-based architectures (Informer, Autoformer and FEDformer), and emerging time series foundation models (PatchTST, TimeLLM, and TimesFM). The authors compare models in terms of forecasting accuracy and practical usability, especially in settings with limited technical expertise. Results show that while deep learning models such as PatchTST perform best overall, simpler models can still offer reliable predictions with minimal setup. Large language models (LLMs) show potential but suffer from computational inefficiency and “pattern deterioration”. Ultimately, the study concludes that robust yet easy-to-use forecasting tools are essential for real-world agricultural applications, where automation and low maintenance are critical.

## 1. Introduction

Time series analysis is an important research field [1]. Since time series arise whenever information is connected over time, time series analysis has become ubiquitous in many data-driven application domains. From analyzing sensor data to understanding financial data, time series analysis has adopted various machine learning methods. Among the many applications of time series analysis, time series forecasting, which aims to predict future values from present and past values of a time series, has become a frequently encountered operation of predictive analysis. This challenging operation has been tackled by many different approaches, from arithmetic algorithms to deep learning models.

In this paper, we focus on the KINLI project, which aims to apply machine learning along the supply chain of the meat industry, from the hatching, nursing and feeding of animals, to the analysis of meat quality and processing. In doing so, KINLI cooperates with turkey farmers to integrate information about turkeys and assemble systems such that farmers are enabled to analyze their turkey’s condition, ultimately aiming to increase animal health by catching issues with the animals early. To this end, KINLI collects sensor data from turkey barns and conducts an initial outlier analysis prior to forecasting. Naturally, forecasting the future values of the collected sensor data would allow KINLI to assess arising issues with the turkey population ahead of time. But creating meaningful forecasts in this field proves to be a challenge. Though the KINLI project has compiled a dataset of sensor data from turkey populations from multiple different barns, the quality in terms of uncertainty of the dataset is very challenging. Each of these barns varies in multiple aspects—not just in size but also in ventilation systems and sensor implementations. Moreover, sensor failures and system outage occur frequently. This results in a dataset riddled with qualitative issues.

Accurate time series forecasting is vital in the domain of animal farming to detect arising issues early, before the animals are impacted negatively. In the domain of turkey farming, the health of the animals develops so quickly that even a few hours of missing information can have detrimental effects on the herd. By making use of time series forecasting, farmers will be able to detect issues in advance. This paper evaluates a variety of forecasting algorithms on the proposed KINLI turkey dataset. We specifically forgo any data cleaning procedures to instead try to forecast the raw dataset. This is because turkey farmers have little to no expertise in the data science domain, necessitating any technical solution to be near fully automated, down to maintenance, data cleaning, and training of ML models. We also want to make sure that the adoption of new barns and new farmers is possible without a long training and optimization phase. So in the end, the goal is to find a model with no or little adoption needs. Our goal is clearly to create a solution that is practical and easy to use, rather than focusing solely on achieving the most accurate results. Therefore, this paper provides a comprehensive evaluation of multiple forecasting algorithms, from statistical algorithms to complex deep learning frameworks, on the raw dataset. We examine why certain models achieve accurate forecasts despite the dataset’s low quality, while others fall short. We also indicate which changes to the models or the entire process would lead to a better result.

The remainder of this paper is structured as follows: In Section 2 we present a brief overview of time series forecasting and respective approaches. In Section 3 we describe our methods and the used dataset. The resulting findings are presented in Section 4. A detailed discussion of the results of the individual models is presented in Section 5. The impact on the health of turkeys during fattening is discussed in Section 6.

## 2. Materials and Methods

In this paper, we focus on univariate forecasting, which considers the following problem:

**Definition 1.** 
*Given a series of time stamps T, let $X\in R^{1\times T}$ be a univariate time series of numeric values. The univariate time series forecasting problem concerns finding a function or algorithm $f\left(\cdot \right):X\to Y$ where $Y\in R^{1\times \hat{T}}$ such that all time stamps in $\hat{T}$ are temporally located after the time stamps in T and $Y=\hat{X}$, where $\hat{X}\in R^{1\times \hat{T}}$.*

Usually, forecasting is complicated so that finding the right algorithm is difficult and values from *Y* and $\hat{X}$ differ. This is evaluated using error metrics, such as the mean squared error or mean absolute error of all values in *Y* compared to $\hat{X}$.

### 2.1. Forecasting Algorithms

Statistical forecasting algorithms are mathematical methodologies employed to estimate future values or trends by utilizing historical data. These algorithms examine the underlying patterns, relationships, and trends present within the data to generate predictions. Their performance relies on the availability of a comprehensive time series. Notable examples of such algorithms include ARIMA [2] and Prophet [3]. Additionally, these algorithms require periodic retraining, the frequency of which is determined by the temporal granularity of the time series data.

### 2.2. Forecasting with ML Models

Classic machine learning models for time series forecasting like XGBoost [4] and LSTM [5] use a supervised approach to learn the inherent properties of time series for predictions. Because of that, it is difficult for these models to capture the complete length of time series for training and prediction and even sometimes ignore temporal dependencies. These algorithms must be trained once on the time series data, but when this is done, this can be sufficient. Transfer learning is therefore not possible.

#### 2.2.1. Time Series Forecasting Using Transformers

Transformer models are a class of machine learning models that were developed to solve natural language problems, such as translations or named entity recognition. The development of the attention layer [6] allowed these models to succeed in this regard. By scaling up model size and providing large amounts of data to train on, these large language models (LLMs) were able to mostly solve natural language processing. OpenAI developed the first large language models, starting with GPT-2 [7] and upscaling to GPT-3 [8], followed by the release of ChatGPT [9] and GPT-4 [10]. LLMs possess capabilities beyond natural language processing, including general pattern recognition and reasoning abilities [11,12,13,14]. Due to this, LLMs are also being adapted to other fields.

Experiments with transformer models in time series analysis have begun shortly after the release of the architecture. However, they faced two problems that made their use for time series forecasting inefficient. (i) *High memory complexity*: Due to the transformer’s memory complexity of $O(L^{2})$ for an input sequence of *L* many tokens, it is difficult to make proper use of it in time series forecasting. (ii) *Insensitivity to locality*: Due to the transformer having no recurrent features, it is unable to model important information of a time series properly, such as seasonalities.

These issues were first addressed with LogTransformer [15], an encoder–decoder transformer that incorporates an adjusted attention layer that improves the memory complexity to $O(L{\left(\log L\right)}^{2})$, by introducing convolutional kernels inside the layer and introducing a timestamp encoding for time series. This led to a series of improved transformer models made specifically for long-sequence time series forecasting. Informer [16] improved the memory complexity to O(LlogL). Autoformer [17] introduced time series decomposition strategies into the model that better provide information about the time series to the models. Finally, FEDformer [18] improved the memory complexity to O(L), with the following improvements:

1. Mixture-of-experts seasonal-trend decomposition (MOE Decomposition): This uses a set of filters with different sizes for multiple trend components from the input, combining them to create a final trend. This replaces the normal transformer’s feed forward layers.
2. Frequency enhanced block: This Fourier transforms the time series to then select random components which are transformed back. This is used at the beginning of both the encoder and decoder.
3. Frequency enhanced attention: This Fourier transforms the input and randomly selects components for the attention computation. This replaces cross-attention.

More improvements in time series forecasting with transformer models include the following: not yet established encoder–decoder transformers [19,20], encoder models [21], and decoder models [22], as well as improvements in model training with different time scales [23] and forecasts with exogenous variables [24].

Beyond these forecasting models, the attention mechanism has been studied for multiple time series analysis tasks independently, such as next frame prediction [25], univariate time series forecasting [26], forecasting with different window sizes [27], and as components in non-transformer networks [28,29].

#### 2.2.2. Time Series Forecasting Using Foundation Models

The transformer models previously described are focused on being as compact and efficient as possible, but they need to be trained on the specific time series that is the objective for forecasting. With the emergence of LLMs, however, the question for a time series foundational model arose, a pre-trained model that can perform any time series analysis task without the explicit necessity to be trained or fine-tuned in the conventional way. However, establishing a time series foundational model faces multiple challenges: (i) **Cross-domain differences**: Time series data are heterogeneous in terms of dimensions and domain specific characteristics. (i) **Language and prompt barrier**: Using pre-trained LMs for time series analysis poses challenges, as the language of the LM is not directly adapted to the time series domain, and the effectiveness of prompts additionally requires domain-specific information. (i) **Generalization conflicts with specificity**: A foundational model is supposed to leverage its generalized knowledge of time series for any given specific time series, where it exhibits issues with identifying and analyzing domain-specific information.

An example of domain-specific challenges is research into forecasting the stock market, which is highly dependent on news sources relevant to the stock. Here, LLMs are frequently used for sentiment analysis of said news articles [30,31,32]. Another use is in the task of modeling and describing stocks [33,34].

This led to research on the creation of time series foundational models [35,36,37,38]. But these efforts were limited due to the low availability of high-quality time series data to train such models on. TimesFM [39] is the first time series foundational model trained purely on time series data. Another approach involved utilizing existing foundation models, which showed first promising results. Utilizing GPT-2 and BERT attention blocks and solely training the embeddings and feed-forward layers on the time series achieves competitive forecast accuracy [40]. PatchTST [41] introduced the idea of patching the time series. By splitting the time series into multiple smaller patches, they were able to enhance the ability of the LLM to process the entire time series without requiring massive computational and memory resources. LLM4TS [42] freezes the attention and feed-forward layers of the LM but still needs to fine tune the other layers inside the model to reach adequate forecasting accuracy. Time-LLM [43] on the other hand demonstrates that adapting existing LMs for time series forecasting without any fine-tuning is possible by using an elaborate patch reprogramming of the numerical values to allow the LM to obtain a textual representation of the time series patches. Unitime [44] further refines the patching by creating a time series tokenizer and proposing a Language-TS transformer which combines the forecasting prompt with the tokenized time series data, which is further refined with dynamic prompt adaptation in Time-FFM [45], with further improvements to time series tokenization by Chronos [46].

However, all previously described approaches use patching and additional strategies to adapt time series to LMs. When using LLMs, it has been shown that it is possible to simply feed the time series to the LLM and achieve high forecast accuracy. Purely prompt-based time series forecasting has been researched with LMs such as T5, Bart, and RoBERTa [47]. LLMTime [48] then used GPT-3 [8] and LLaMA-2 [49] as the basis of their forecasting model, but further research has remained sparse.

## 3. Test Setup/Method Description

### 3.1. The KINLI Dataset

The KINLI dataset comprises multiple sensor values originating from turkey farming barns. It is part of a system that monitors the state of each barn, with forecasts to detect possible issues early. This dataset encompasses all challenges that can be found in real-world dataset. There are sensor errors where no values are being recorded and outlier values due to things like the farmer cleaning the system without turning off the sensor. Furthermore, the timestamps for each sensor recording are inconsistent, with a value recorded roughly every 10 min. This has to do with the way in which the data is collected. It is not read via an interface but via a web scraper, which has a different execution time for each cycle and therefore cannot achieve an exact interval. Direct access to the database is not possible. As each barn was added to the system sequentially, none of the barns have the same number of sensor values recorded. Older recordings are also even less frequent, sometimes only once or twice per hour. The dataset contains 14 months of data from 20 different turkey pens. The values are reset to zero after each day and the time series starts again from the beginning. The data comes from stables with animals of the BUT 6 variety and is recorded in the stable from week six to week 22. Only roosters are used for fattening.

**Figure 1:**
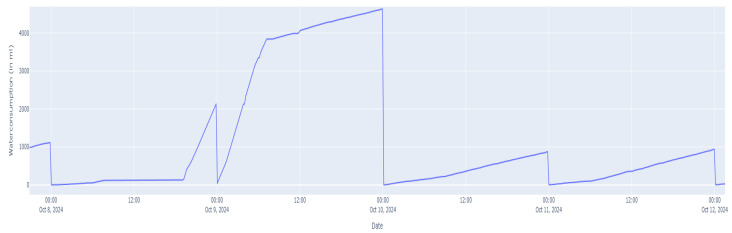
Visualization of the KINLI dataset.

This dataset is intriguing to use precisely because of its imperfections, a snippet of which can be seen in Figure 1. Common benchmarks for forecasting algorithms are datasets with very few errors, especially not of this kind. This will give an overview of how these forecasting algorithms perform in a real-life setting.

Another concern is the amount of pre-processing necessary to make proper forecasts on this dataset. Agriculture and animal farming is not a widely digitalized industry, at most relying on proprietary software that does not offer analytical capabilities and suffers from extreme vendor lock-in. Because of that, we want to try to use as little pre-processing as possible so that the setup and deployment of the forecasting solution are as easy as possible for turkey farmers, even without extensive knowledge about machine learning and data science. In addition, every barn is different with respect to structure, technical equipment, location, and climate, and every farmer has a different approach. Each turkey fattening cycle is also different, and the cycles for each barn do not start at the same time and are different in length.

Regarding the task of time series forecasting, we consider the following values:

**Water/Day** tracks the amount of water distributed to turkeys in a barn. This sensor accumulates until a final value is reached at 23:59:59 and resets for the next day. Normally it accumulates slowly until 06:00:00. Then the turkeys wake up and start drinking, which leads to a steeper rise until 23:00:00 when the turkeys go to sleep. This value is calculated by dividing the total consumption by the number of animals present.
**Food/Day** tracks the amount of food distributed to turkeys in a barn. This sensor also accumulates until a final value is reached at 23:59:59 and resets for the next day. Unlike the water sensor, the feed pump only runs a few minutes per day, meaning the sensor data remains unchanged for long times and then shoots up as the food pump runs for a few minutes to refill the food for the turkeys. A critical time is when the turkeys wake up and start eating food, between 06:00:00 and 08:00:00. This value is calculated by dividing the total consumption by the number of animals present.
**Water/Food** is the ratio of water to food consumed by turkeys, based on the previous two sensor values. This indicator is vital to detecting diseases in turkey, as a sickly herd will stop eating food.

### 3.2. Experiment Set-Up

The framework from [18] was used for the training and inference of the models. For all the deep learning models a GPU Cluster via SLURM was used for the statistical and ML models. The Inference for the Non-Open-Source Models was performed via the according APIs from the vendors. The mean squared error and mean absolute error were used as metrics. The experiments were carried out several times and the respective mean value was calculated. Data transformation was only carried out to the extent that it was brought into the appropriate form for the respective model. The steps for this are explained in the model section. Strategies for the LLM models can be found in Appendix A. Hyperparameters for the models are in Appendix B. If a batch size can be used, it is 32.

### 3.3. Considered Algorithms and Models

**Statistical algorithms**: ARIMA [2] and Prophet [3] are frequently used for time series forecasts and often show better results than deep learning models. SARIMA was not chosen, as there are no fixed lengths for individual cycles or seasons. The models do not require any scaling of the data. To perform the tests, it is necessary that the model is refitted after a prediction step. This makes the tests significantly longer.
**Machine learning algorithms**: XGBoost [4], a Gradient Boosting Model, was chosen because it is easy to implement and offers fast training and inference times. It is frequently used in industry for time series, with good results. The model does not require any scaling of the data. A sliding window was used to transform the data to make them accessible for XGBoost. This allowed the time series to be presented as tabular data. A multi-output regressor from [50] was also used to predict several time steps at once. This was not necessary for the EOD forecast.
**Deep neural networks**: We use three types of simple linear models as a baseline for deep learning algorithms. These are called ‘Linear’ which is just a single linear layer, ‘NLinear’ which applies normalization to the linear layer, and ‘DLinear’ which uses time series decomposition and a moving window trend, similar to Autoformer [17], before the linear layer.
**Recurrent neural networks**: LSTM [5] models can recognize long-range dependencies and with automatic feature extraction, no pre-processing other than scaling is necessary. Before the advent of Transformer models, LSTM models were the industry standard for sequential data.
**Transformer models**: When it comes to transformer models, we first use a basic transformer model [6], and then three of the specialized forecasting models: Informer [16], Autoformer [17], and FEDformer [18].
**Time Series Foundation Models**: We test PatchTST [41], which introduces patching, as well as TimeLLM [43], which combines patching with reprogramming to allow forecasting with proprietary LLMs. And at last we use TimesFM [39] as the first time series foundational model trained purely on time series data.
**LLM forecasting models**: Following the results of LLMTime [48], we adapt their results to our tests by using smaller open-weight LLMs, not larger than 10 billion parameters. In addition to that, we also implement some prompting strategies for time series forecasting with LLMs, instead of only feeding raw values into the LLM. The strategies used can be found in Appendix A. We focus on smaller LLMs due to the fact that these models, were they to be implemented in KINLI, would have to be run locally at the farmer’s own server infrastructure, which is not particularly powerful. They are also more cost efficient due to this fact. We use Falcon-7b [51] because it is a small LLM that is not remarkable, maybe even outdated, to compare against more advanced LLMs. The other LLMs we test are from the LLama series of LLM models [49,52], namely LLama-3-8b, LLama-3.1-8b, LLama-3.2-1b, and LLama-3.2-3b [53] since they are known for their good performance for their size. We only use base models, as they did not receive extensive RLHF, which should have a positive impact on performance [48].

### 3.4. The End-of-Day Forecast

To be able to properly detect problems in the barn and help immediately, we aim to forecast the end-of-day value (eod value). By comparing the forecast of each day over the entire growing cycle (cycle value) of the turkeys against the forecast of the eod value of the current day using today’s sensor data, it is possible to detect possible errors if the eod value is too far below or above the cycle value. And thus, we have the opportunity to recognize problems or illnesses as early as possible, possibly even before they can occur. What is important here is that it is not necessary to make an exact prediction; what is more important is how large the deviation will or can be. This test concerns forecasting the eod value using the available sensor data at present, from the current day and forecasts the next values until the eod value. We consider the tests of forecasting the end-of-day value from (1) 22:00:00 (2) 18:00:00, (3) 12:00:00, and (4) 08:00:00, as well as (5) the critical time when the turkeys are just waking up, forecasting the 08:00:00 time using all values up to 06:00:00.

### 3.5. Long-Sequence Forecast

After evaluating these forecasting algorithms on the end-of-day test, we choose promising algorithms to perform a series of long-sequence forecasts, where the sequence length is increased to take multiple days of sensor values into account before making the end-of-day forecast. Each of these tests aims to forecast the end-of-day value from 08:00:00, as this is the most challenging end-of-day forecast.

## 4. Results

**Figure 2:**
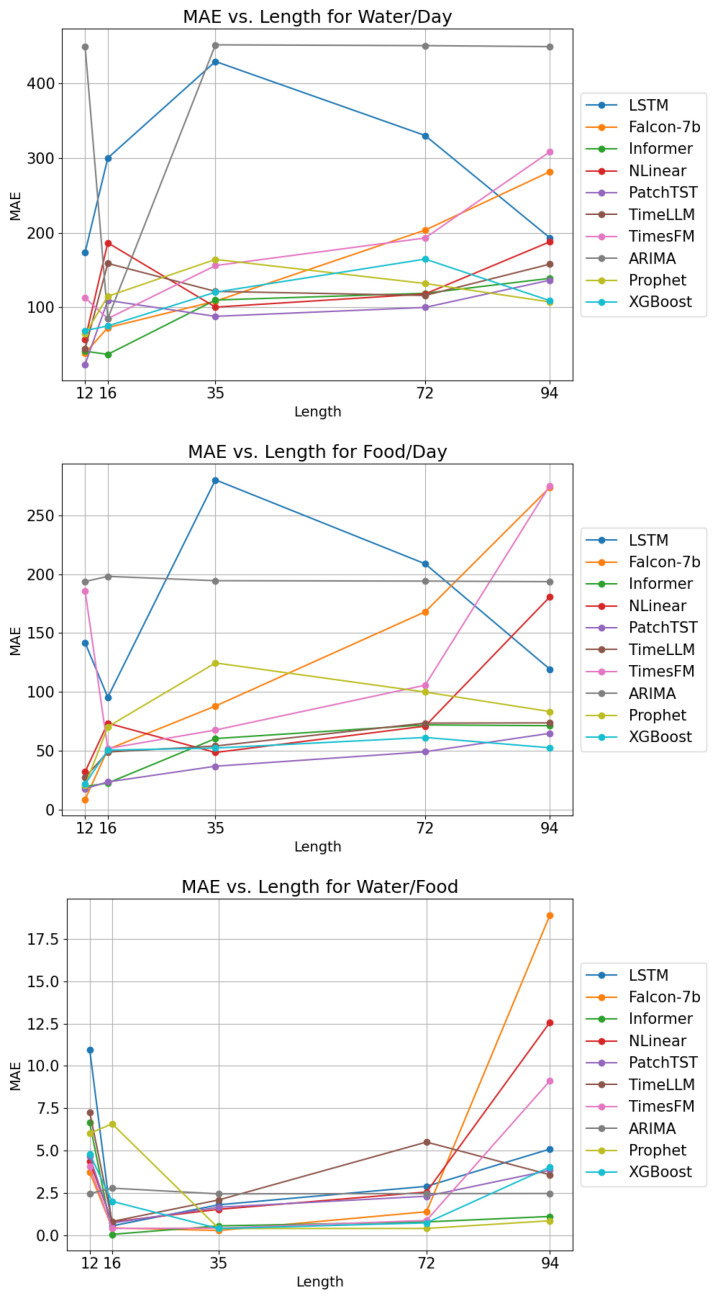
Forecast MAE ordered by forecasting length, with 12 corresponding to the 06:00:00 to 08:00:00 test, 16 being the 22:00:00 to eod test, 35 18:00:00, 72 12:00:00, and 94 08:00:00.

This section presents the results of our tests. The following Figure 2 shows the result of the different models for each of the forecasting horizons and the different prediction values. The results for all models can be found in Appendix B.

**Figure 3:**
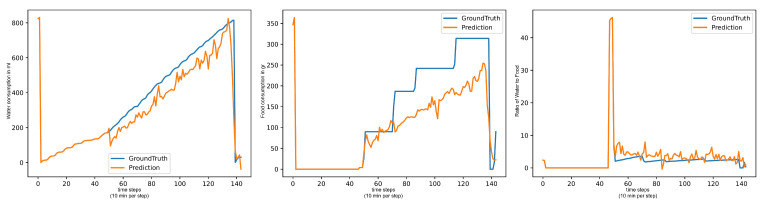
Forecasts of informer: water per day in ml (**left**), food per day in gr (**center**), water per food (**right**).

Machine learning and deep learning models that need to train on the dataset all have issues with outliers impacting their forecasts. As a result, even on a seemingly simple target such as (Water/Day), where the sensor value each day roughly follows an upwards trend, these models forecast big jumps between each consecutive value, even if the overall trend of the forecast is correct. Models with better metrics usually forecast less severe jumps between values. Examples can be seen in Figure 3. Statistical models have problems with the first predictions, as they always make a very large jump, up or down. However, they then approach the ground truth curve again in phases, only to be completely wrong at the end of the day by ending the day too early.

The linear models serve as a baseline for deep learning model performance. As they simply consist of a single linear layer, we consider any more complex deep learning model that has worse accuracy to be a failure. On all three basic tests, this is only the case for TimeLLM. All other models are better. On the end-of-day tests, linear models improve comparatively as the forecasting length increases. The normalization linear model (NLinear) performs particularly well in all three end-of-day tests. However, in the test from 06:00:00 to 08:00:00, the normalization linear model is worse.

The specialized transformer models all face a noticeable forecast accuracy issue on the unscaled dataset, which is more extreme with the vanilla Transformer and Informer. On the unscaled dataset, these models are terrible, while on the scaled dataset, these two models are among the best models of each test. Other models do not show such a significant difference between scaled and unscaled versions. The sensitivity to scaling is inverted on the (Water/Food) forecasting test. Here, these models perform better on the unscaled dataset.

**Figure 4:**
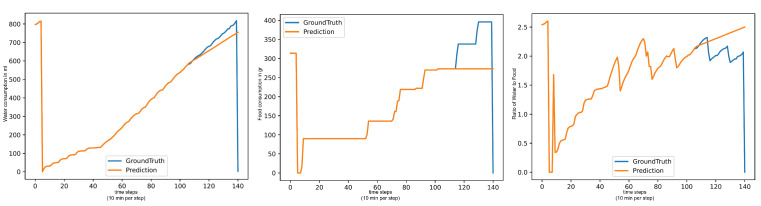
Forecasts of LLMs: Water per day in ml (**left**), Food per day in gr (**center**), Water per Food (**right**).

The LLMs performance varies heavily between the different tests. In the basic test, the LLMs achieve good metrics on all three targets. On the end-of-day tests, the LLMs still perform decently on the 18:00:00 test but drop sharply for the 12:00:00 test, the 08:00:00 test, and the critical-time test. On the (Water/Day) forecast, the LLMs show seemingly normal performance but all have highly similar metrics on (Food/Day) and (Water/Food). This is due to an issue with pure LLM forecasting that we call “Pattern Deterioration”. LLMs have a habit of simplifying the forecasting pattern down to producing a flat line. Due to the shape of the (Food/Day) data, this process is instant, which is why all these models have the same metrics. On (Water/Food) the deterioration still occurs, but since the values of this target always remain around the same value, a flat line after a few values is not a bad forecast, while on (Water/Day), the pattern deteriorates into an upward trend line, which is why the LLMs perform decently there. This is shown in Figure 4.

One unique issue of the LLM-based forecasting is the topic of their compute time. During testing, it has been shown that forecasting the time series one value at a time, up to the forecasting length, results in the highest forecast accuracy. However, that means that the computation time scales with the forecasting length, while these models already have the highest inference time of all tested models. All other models are much faster and less resource hungry in comparison.

TimeLLM performs worse than the direct LLM forecast with the same model on the basic forecast with all models tested. This is peculiar because TimeLLM uses patch reprogramming to allow the LLM to obtain a text representation of the time series to improve accuracy and avoid issues such as pattern deterioration. On the end-of-day tests, the TimeLLM performance improves comparatively to the LLMs due to training, and on the 12:00:00 and 08:00:00 tests, it even reaches a performance in the higher middle field with GPT-2 as the backbone. We surmise that TimeLLM is sensitive to the models used with it and cannot properly use newer LLMs, which is why performance is best with GPT-2. In addition to this, TimeLLM is scaling sensitive. Although the difference is not as severe as with the specialized transformer models, TimeLLM shows the same preference for scaling, and the same inverse preference for unscaled data on (Water/Food).

PatchTST on the other hand performs well in all tests, being among the best performing models. It is also not scale sensitive.

**Figure 5:**
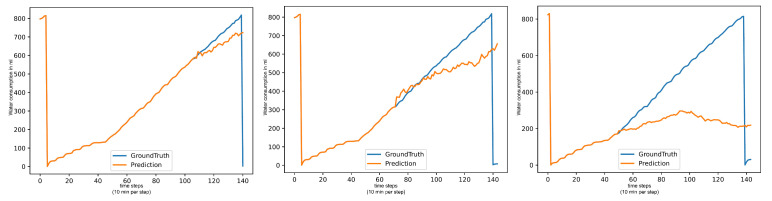
Forecasts of TimesFM on water per day in ml: 18:00:00 (**left**), 12:00:00 (**center**), 08:00:00 (**right**).

The performance of TimesFM is initially very good but drops heavily on the end-of-day tests. Being a model that did not receive fine-tuning on the dataset, the model’s forecasting output is heavily impacted by the length of the input sequence. Therefore, as the timestamp gets closer to 00:00:00 and the forecasting length increases, TimesFM has a particularly hard performance drop. The (Water/Day) end-of-day test from 08:00:00 in Figure 5 showcases the issue with TimesFM particularly well, as it is not able to pick up on the increased water intake from the waking turkeys, forecasting a lower rise in line with values from when the turkeys were still sleeping.

### Long Sequence Forecasts

To gain further insights into the performance of the forecasting algorithms on our dataset, we performed a series of long sequence forecasts on (Water/Day), with the sequence lengths being 256, 512, and 1024 in 10 min time steps. We were especially interested in the performance of models that did not train on the data, such as the direct LLM forecasts and TimesFM, and compared them against the performance of trained models. Table 1 shows an overview of the results of these forecasts.

**Figure 6:**
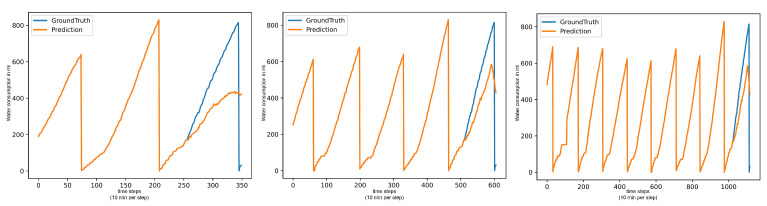
TimesFM long sequence forecasts for Water/Day in ml for 08:00:00 to 24:00:00 are able to better identify the shape of the dataset, but still fail to improve accuracy.

The models that train on the entire dataset show no significant improvements in their metrics. For the models that do not train, TimesFM and the LLMs, the performance does increase significantly but only up to a limit. Figure 6 shows that TimesFM only improves the MAE to 241.0 on the sequence length of 256, but further increasing the sequence length does not produce any further improvements. Although this is a significant improvement over performance in the end-of-day test, where the MAE is greater than 300, it still is among the worst models of these tests.

**Figure 7:**
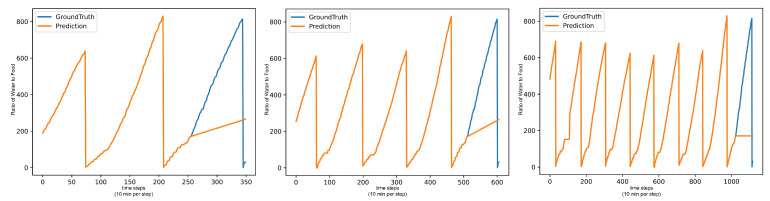
LLM long sequence forecasts for Water/Day in ml for 08:00:00 to 24:00:00 still show pattern deterioration.

Figure 7 shows that LLMs also show a significant performance increase with the added sequence length but are not able to catch up to the trained models either. The additional sequence length also does not reduce the previously described issue of pattern deterioration.

## 5. Discussion

In this section, we will offer some discussion of the results of our forecasting tests.

### 5.1. Issues with LLMs in Time Series Forecasting

**Figure 8:**
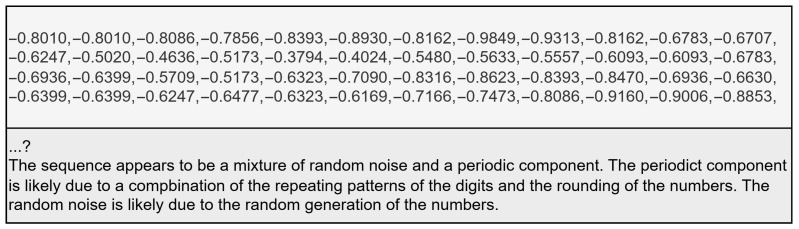
Example of unrelated output with input (**top**) and output (**bottom**).

**Figure 9:**
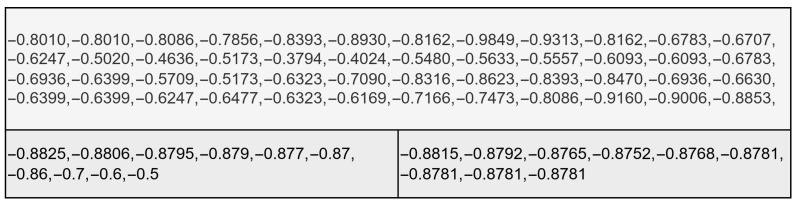
Examples of output deterioration with input (**top**) and precision deterioration (**left**) and pattern deterioration (**right**).

When we adapted these LLMs for time series forecasting, we identified the following reoccurring issues:

**Unrelated output**: This refers to the model output being unrelated to the given prompt. Usually, this results in the output containing either no usable numeric values at all or having noticeable output deterioration to the point of making the forecast meaningless. An example is shown in Figure 8. The risk of unrelated output increases with each non-numeric token in the input prompt; therefore, the most successful forecasts include no text prompt but only time series values. We also found that LLama models generate unrelated output more often than Falcon-7b.
**Precision Deterioration**: This refers to the phenomenon that the output values become imprecise with increasing forecasting horizon. As shown in Figure 9, after the model generates some values with the correct precision, it then starts reducing the precision and never recovers, reducing the output to a simple set of single-digit numbers. To combat this, we filter the model’s output to only accept values that have the required precision or higher and discard any generated lower precision values. This leads to a higher amount of forecasts failing, like rounding to 12 digits or keeping the original 16 digit precision.
**Pattern Deterioration**: As mentioned previously, this refers to the model output values presenting less and less complex patterns with increasing forecasting horizon, until it settles on a single value that is repeated over and over again. This usually happens quickly; in just four to five values, the LLM reduces the pattern to a constant but this can be faster if the time series already contains repeating values. This is shown in Figure 9. Pattern deterioration remains the key challenge open-weight LLMs of this size face when forecasting a time series directly.

### 5.2. Issues with ARIMA

ARIMA does not perform well in all tests; this is because the data does not represent good statistical patterns. Seasonal and recurring patterns are not strongly represented in the data, so this leads to problems. The fact that the intervals between the individual time stamps are not always the same also causes problems here. It is also problematic that the performance of ARIMA depends very much on the hyperparameters, which were not tested too extensively in this scenario. Even if the tests performed well, a major problem here would be the constant training of the model on the limited data available.

### 5.3. Issues with Prophet

Prophet performs relatively well on the data, but problems arise with longer forecasting lengths. For use, Prophet has the problem that it always has to be completely retrained on new data; retraining is not possible in our scenario. We want to ensure that the model works out of the box without retraining or refitting to a different barn.

### 5.4. Issues with XGBoost

The main problem with XGBoost is that the hyperparameters are very important for this model and a lot of optimization is required. Pre-processing the data so that they can be used for a time series prediction is also very time-consuming.

### 5.5. Issues with LSTM

There are relatively little data for LSTM such that it can be trained well. Another problem for the LSTM is that the models often require hyperparameter tuning, which was not performed in detail in this scenario due to the limited number of pre-processing steps. The training of the model is also very slow and resource intensive.

### 5.6. Issues with Specialized Transformer Models

The interesting observation about the specialized Transformer models is that the performance of the newer FEDformer [18] and Autoformer [17] is usually worse than that of Informer and the regular Transformer. We surmise that the time series decomposition introduced by Autoformer is at fault, as FEDformer further enhances this component. These models were optimized for memory complexity by observing patterns in the attention layer when forecasting and enhancing attention with these patterns. But these patterns do not appear when forecasting our dataset without pre-processing, leading to information loss and therefore lower forecasting accuracy compared to Informer or even just the vanilla Transformer model. All transformer models exhibit ’erratic’ forecasts due to the high amount of inaccuracies and anomalies in our dataset.

### 5.7. Issues with TimeLLM

The performance of TimeLLM [43] is in contrast to PatchTST [41], which performs well on all tests. This demonstrates that patching the time series is not the issue and perfectly suited for forecasting messy data like this. Instead, TimeLLM performance can be explained by the two main differences from PatchTST. The model’s reliance on pre-trained LLMs that are not directly adapted to time series forecasting is the main cause of the discrepancy in performance, as it is in line with the raw LLM forecasting performance. The second issue is the patch reprogramming used to adapt the patches to the LLMs which understand the text best. Patch reprogramming is successful in the sense that it eliminates the issues that LLMs face when forecasting, such as pattern deterioration. However, the adaptation leads to a worse performance than the LLMs in cases where the simpler pattern of the LLM forecast is fitting, such as (Water/Day) or (Water/Food). The comparative improvement in performance over LLMs in longer forecasting lengths can be attributed to the fact that this model still trains on the entire dataset, eliminating the key weakness of short sequence lengths.

Due to the overall bad performance and training requirements of TimeLLM, we would not recommend using this model in production at this time.

### 5.8. Issues with TimesFM

With TimesFM [39] we observe the main issue of the short sequence length of the end-of-day tests. Since the model does not train on the dataset, the performance is directly related to the amount of information put into the model, meaning that the short sequence length leads to poor forecast accuracy. The test of 06:00:00 to 08:00:00 demonstrates that TimesFM is more impacted by the length of the input sequence than the forecasting length, as performance remains poor.

### 5.9. Possibilities with Pre-Processing

Significantly better results could be achieved by pre-processing the data. However, the authors decided against this because the aim was to test the models virtually out of the box. A farmer should later have the option of adding a new barn or generally using the system without having to make any major preparations. Data availability in this area is also not particularly high, which means that training models or pre-processing based on metrics is not possible.

### 5.10. Fine-Tuning of LLM Models

Fine-tuning the LLM would significantly improve its results but at a high cost in terms of resources. However, it remains questionable whether an individual farmer would be able to fine-tune such a model using their existing data. Looking at the entire dataset across stables, it is certainly possible to achieve better results. However, training would contradict the original idea that no or only minor adjustments should be made to a model.

## 6. Conclusions

### 6.1. Result Discussion-Impact on Poultry Health/KINLI

Achieving the daily dose is very important for turkey fattening; larger deviations can be indicators of disease, or poor water or feed quality. The earlier it becomes apparent that the daily dose cannot be reached, the faster countermeasures can be taken. In the fattening of turkeys, 5–6 h are crucial here to react; during this period, sick animals can possibly be saved. The earlier the daily dose can be predicted, the faster any diseases can be recognized. Any problems with the water or feed supply can also be identified. It can also be determined whether the procedure needs to be changed for a fattening cycle because the animals can react differently to feed or water quality than in previous cycles. However, in order to achieve this, the farmer must have an approximate forecast of the animals’ consumption as early as possible. Ultimately, this ensures that the animals remain healthy and that turkey fattening can be carried out sustainably in terms of resource consumption. In the end, it is not crucial that the prediction is as accurate as possible but that it can be applied quickly and easily and can also be applied to new barns without starting a long data collection process and pre-processing it accordingly afterwards. At this point, however, it must be clearly stated that testing across different stables and different fattening cycles is still pending in order to ultimately ensure that farmers can work with it, and that the health of the animals is also improved as a result.

### 6.2. Conclusions

We were able to identify the best models for our application, which are Informer and PatchTST. Although XGBoost and Prophet also offered good forecast accuracy, the required hyperparameter tuning and regular retraining requirements make them unattractive in the field of poultry farming, where there is usually very little data to train or adapt time series forecasting models. Models that did not train on the dataset, such as LLMs and TimesFM, were easier to use but failed to achieve satisfactory forecast accuracy. TimeLLM did reach good accuracy with GPT-2 as the backbone but was more costly to train. After evaluating these models on the dataset with these tests, we came to the conclusion of focusing on PatchTST in the production environment. With this we will be able to make forecasts with satisfactory accuracy to help detect problems in the poultry pen ahead of time, without a long phase in the data collection, pre-processing, and adopting phase. We have demonstrated that, with very little effort in terms of data preparation and training, it is possible to use LLM models to forecast consumption data in turkey farming. However, the results are not yet accurate enough to make completely precise predictions. The initial results are good enough to test them in practice. The predictions are made available via a REST API and incorporated into the visualizations of the sensor values. This makes them easily accessible to farmers. At the same time, the predicted value is compared with a threshold value for the respective fattening period, and if it overly exceeds or falls below this value, an email is sent to the farmer.

Further research plans include testing new smaller models that have been published in the meantime, as well as retraining any small LLM models in order to achieve better results. There are also plans to train smaller models in order to obtain a kind of foundation model, which would make it much easier to adapt and apply to new pens.

### 6.3. Concluding Remarks and Novel Contributions

In conclusion, this work contributes to the research on time series forecasting by systematically evaluating a broad spectrum of forecasting paradigms—from classical statistical models to modern deep learning and foundation architectures—on sensor data collected from real turkey barns. This setting represents a highly complex and practically relevant application domain, where sensor failures, irregular data intervals, and environmental variability pose significant challenges for predictive modeling. By focusing on poultry health monitoring, the study demonstrates how time series forecasting can support early detection of anomalies and improve animal welfare in modern livestock management systems. Furthermore, we empirically observe a degradation effect in LLM-based forecasting, where large language models tend to simplify temporal dynamics into flatter trajectories over longer forecast horizons—a behavior that may limit their applicability for real-world longtime monitoring tasks. Overall, the findings highlight both the opportunities and the limitations of applying foundation models in agriculture, and provide a foundation for future research on data-driven animal health monitoring under realistic field conditions.

## Figures and Tables

**Table 1:** Test results of long sequence forecasts on 08:00:00 to end-of-day (Water/Day) for select models.

Dataset	Sequence Length	Forecasting Length	Model	MSE	MAE
Water/Day	50	94	LLama-3.2-3b	144,172.3	246.67404
Water/Day	50	94	Informer	53,442.754	138.82674
Water/Day	50	94	PatchTST	56,044.457	136.47639
Water/Day	50	94	TimeLLM-GPT-2	59,466.441	157.79073
Water/Day	50	94	TimesFM	167,285.69	308.22034
Water/Day	256	94	LLama-3.2-3b	123,470.1	196.3396
Water/Day	256	94	Informer	49,009.93	137.2414
Water/Day	256	94	PatchTST	43,613.63	119.1713
Water/Day	256	94	TimeLLM-GPT-2	53,003.62	143.8254
Water/Day	256	94	TimesFM	106,913.9	241.1781
Water/Day	512	94	LLama-3.2-3b	124,592.6	201.5027
Water/Day	512	94	Informer	52,098.79	149.1073
Water/Day	512	94	PatchTST	50,142.46	143.5388
Water/Day	512	94	TimeLLM-GPT-2	70,224.34	190.5589
Water/Day	512	94	TimesFM	106,130.4	243.7242
Water/Day	1024	94	LLama-3.2-3b	94,902.53	175.6282
Water/Day	1024	94	Informer	51,740.08	149.0719
Water/Day	1024	94	PatchTST	48,542.24	139.8018
Water/Day	1024	94	TimeLLM-GPT-2	48,614.13	142.5252
Water/Day	1024	94	TimesFM	106,130.4	243.7242

## Data Availability

The data presented in this study are available on request from the corresponding author due to data protection reasons and due to business confidentiality.

## Appendix A. Prompt Strategies for LLM Based Forecasts

We used an experimental approach that allowed us to test a variety of possible prompt and data injection strategies with the LLM. With this setup, we tested a variety of prompting strategies to see how they would perform.

### Appendix A.1. Spacing

Introducing possible spacing between individual digits of input values, as suggested in [48] to have the tokenizer tokenize each digit individually.

**Table A1:** Spacing strategies.

No Spacing	Spacing
0.856776	0 . 8 5 6 7 7 6

We were unable to corroborate the claim that added spacing for certain tokenizers increases performance. Instead, forecasting performance was always worse when spaces between the digits were added.

### Appendix A.2. Input Length

Shortening the input length down from the original sequence length to half or a quarter of that. This did not have any positive effects, and we recommend always giving the LLM as much information as possible.

### Appendix A.3. Data Format

The time series is given to the LLM either as a closed list of values, enclosed by brackets, or as an open list that ends in a comma or a tabular view with timestamps, which is intended to encourage the LLM to continue generating numbers.

**Table A2:** Data formats.

Open List	Closed List	Tabular
...,0.856776,0.867993, 0.882301	[...,0.856776,0.867993, 0.882301]	...,12:34:26; 0.867993, 12:44:51; 0.882301

### Appendix A.4. Rounding

This entails rounding the input down from 16 digits to either 12, 8, 7, 6, 4 or 2 digits after the decimal point. We found that not rounding the values leads to failure due to precision deterioration. Depending on the LLM, the amount of rounding necessary may vary, but for open weights LLMs in the range of 7b parameters, the best forecasting performance was reached on both 7 and 6 digits.

### Appendix A.5. Integer Transformation

Removing the decimal point to make the numbers simple integers. This uses two possible strategies, either removing leading 0s or keeping them. Transforming the numbers and removing leading 0s encourages precision deterioration, usually leading to a failure to produce output values with the proper precision. Between keeping the original value and keeping leading 0s, the performance depends on the original values. For values that frequently have two or more digits in front of the decimal point, no integer transformation is recommended, but for values that only have a single digit before the decimal point, such as most scaled datasets, the simple integer transformation is better.

**Table A3:** Integer transformation.

Original Value	Simple Integers	Complex Integers
0.985543	0985543	985543
1.112436	1112436	1112436

### Appendix A.6. Prediction Task

This is providing either only the data as is without a given task, or providing a simple task description. These might either be to predict the next 16 steps at once, or predict missing steps until the prediction length is reached, or at last only predict the single next point. All tasks follow the prompt-as-prefix format that has been shown to be effective [43]. Our prompts can be found in Table A4. Introducing a task prompt has different effects on the observed LLMs. For Falcon-7b, the prompts that ask the LLM to always predict the next 16 values, regardless of currently missing values, result in slightly improved performance over no task prompt, while both other tasks are slightly worse. For Falcon-7b the differences between the prompts were very minimal. With LLama-3-8b, using no prediction task prompt has the best performance, then predicting the next value and predicting 16 always are similar, and predicting the next missing values is worst. The differences in performance are much larger for LLama-3-8b than for Falcon-7b but still very small. Because of this, both models have their best strategies distributed between each prediction strategy, except for missing prediction.

**Table A4:** Prompt strategies used.

Prompt Type	Prompt
None	{data}
Predict 1	Predict the next value of the following numeric sequence: {data}
Predict 16	Predict the next 16 values of the following numeric sequence: {data}
Predict missing	Predict the next {missing} values of the following numeric sequence: {data}

### Appendix A.7. Knowledge Enhancement

Providing simple information about the previous 96 steps provides possible knowledge enhancement. These contain two settings. The first one simply lists all knowledge enhancements we made, the second one only picks some of them. A list of all used knowledge enhancements can be found in Table A6. Full KE and basic KE had negative effects on forecasting accuracy, while minimal KE and no KE have minimal performance differences and are overall the same. The effectiveness of KE on these LLMs is limited due to the previously outlined issue of pattern deterioration.

**Table A5:** List of possible knowledge enhancements, and where they are used.

Information	Used by Full KE	Used by Basic KE	Used by Nimimal KE
Minimal value	yes	yes	yes
Maximal value	yes	yes	yes
Mean value	yes	yes	no
Median value	yes	yes	no
Standard deviation	yes	yes	no
General trend	yes	yes	yes
Variance	yes	no	no
Range value	yes	no	no
25th percentile	yes	no	no
75th percentile	yes	no	no
Mode value	yes	no	no
Kurtosis value	yes	no	no
Skewness value	yes	no	no
Autocorrelation	yes	yes	no
Value count	yes	yes	yes

## Appendix B. Results for the Individual Prediction Tasks

**Table A6:** Best results of model evaluation on raw water per day.

Dataset	Length	Model	Settings	MSE	MAE
Water/Day	16	LLama-3.1-8b	Round7, pred 16a	57,840.102	68.47949
Water/Day	16	LLama-3.2-1b	Round6	58,941.66	64.88672
Water/Day	16	LLama-3.2-3b	Round6, minimal KE	58,611.02	69.29980
Water/Day	16	LLama-3-8b	Round7, pred16a	58,413.547	69.79590
Water/Day	16	Falcon-7b	Round7, pred1	57,588.238	72.88477
Water/Day	16	FEDformer	dm1024 nh8 el4 dl4 df2048 fc3	101,843.289	225.293
Water/Day	16	Autoformer	dm512 nh8 el4 dl4 df2048 fc2	138,818.594	322.072
Water/Day	16	Informer	dm1024 nh8 el4 dl4 df2048 fc3	627,469.875	743.984
Water/Day	16	Transformer	dm1024 nh8 el4 dl4 df2048 fc3	627,754.438	744.168
Water/Day	16	DLinear		81,499.10	194.8440
Water/Day	16	NLinear		77,887.39	186.6709
Water/Day	16	Linear		89,925.68	209.81595
Water/Day	16	PatchTST		35,381.09	109.3663
Water/Day	16	TimeLLM	Llama-2-7b	88,190.01	219.6265
Water/Day	16	TimeLLM	GPT2	56,871.81	160.2766
Water/Day	16	TimeLLM	Llama-3.2-1b	104,556.6	234.1954
Water/Day	16	TimeLLM	Llama-3.2-3b	72,011.2	192.8992
Water/Day	16	TimeLLM	Llama-3.1-8b	59,742.5	167.828
Water/Day	16	TimesFM	no fine tuning	62,865.28	84.95092
Water/Day	16	ARIMA	96, 2, 1	333,688.7494	462.066
Water/Day	16	Prophet	seasonality = daily, mode = multiplicativ	27,151.4590	114.79
Water/Day	16	XGBoost	estimators = 700, depth = 6	34,543.4298	75.05
Water/Day	16	LSTM		132,292.36	299.84

**Table A7:** Best results of model evaluation on raw food per day.

Dataset	Length	Model	Settings	MSE	MAE
Food/Day	16	LLama-3.1-8b	Round6	8853.478	51.54004
Food/Day	16	LLama-3.2-1b	Round6	8853.478	51.54004
Food/Day	16	LLama-3.2-3b	Round6	8853.478	51.54004
Food/Day	16	LLama-3-8b	Round6	8853.478	51.54004
Food/Day	16	Falcon-7b	Round6, pred16a, integers with 0s	8788.886	51.10449
Food/Day	16	FEDformer	dm1024 nh8 el4 dl4 df2048 fc3	4287.314	44.77009
Food/Day	16	Autoformer	dm512 nh8 el4 dl4 df2048 fc3	13,844.835	87.94547
Food/Day	16	Informer	dm1024 nh8 el4 dl4 df2048 fc3	28,238.459	139.49284
Food/Day	16	Transformer	dm1024 nh8 el4 dl4 df2048 fc3	33,873.613	157.26195
Food/Day	16	DLinear		11,288.98	73.32854
Food/Day	16	NLinear		10,931.58	73.36824
Food/Day	16	Linear		12,085.05	77.60903
Food/Day	16	PatchTST		1343.072	23.36775
Food/Day	16	TimeLLM	Llama-2-7b	8176.939	66.14028
Food/Day	16	TimeLLM	GPT2	5703.720	51.06726
Food/Day	16	TimeLLM	Llama-3.2-1b	9821.357	68.67682
Food/Day	16	TimeLLM	Llama-3.2-3b	18,011.233	105.4492
Food/Day	16	TimeLLM	Llama-3.1-8b	14,496.47	90.37284
Food/Day	16	TimesFM	no fine tuning	9961.349	55.96097
Food/Day	16	ARIMA	96, 0, 6	61,003.7543	198.2594
Food/Day	16	Prophet	seasonality = daily, mode = multiplicative	8190.93964	70.3218606
Food/Day	16	XGBoost	estimators = 700, depth = 6	10,768.6810	50.45
Food/Day	16	LSTM		15,114.26	95.41

**Table A8:** Best results of model evaluation on raw water per food.

Dataset	Length	Model	Settings	MSE	MAE
Water/Food	16	LLama-3.1-8b	Round6, integers with 0s	0.708403	0.566563
Water/Food	16	LLama-3.2-1b	Round6	0.685307	0.557363
Water/Food	16	LLama-3.2-3b	Round7, pred 1, minimal KE	0.668494	0.560215
Water/Food	16	LLama-3-8b	Round6, pred1, minimal KE	0.730340	0.556641
Water/Food	16	Falcon-7b	Round7, pred16a	0.441695	0.437510
Water/Food	16	FEDformer	dm512 nh8 el2 dl1 df2048 fc3	1.869564	1.134118
Water/Food	16	Autoformer	dm512 nh8 el2 dl1 df2048 fc3	1.076691	0.827988
Water/Food	16	Informer	dm512 nh8 el4 dl4 df2048 fc3	0.218783	0.327771
Water/Food	16	Transformer	dm512 nh8 el4 dl4 df2048 fc3	0.098450	0.242006
Water/Food	16	DLinear		0.869079	0.756642
Water/Food	16	NLinear		0.949909	0.789085
Water/Food	16	Linear		0.770473	0.709996
Water/Food	16	PatchTST		0.951654	0.725133
Water/Food	16	TimeLLM	Llama-2-7b	1.469922	1.047710
Water/Food	16	TimeLLM	GPT2	1.046001	0.860559
Water/Food	16	TimeLLM	Llama-3.2-1b	1.053665	0.870521
Water/Food	16	TimeLLM	Llama-3.2-3b	1.074678	0.890120
Water/Food	16	TimeLLM	Llama-3.1-8b	1.848694	1.189474
Water/Food	16	TimesFM	no fine tuning	0.395222	0.404078
Water/Food	16	ARIMA	96, 0, 6	31.72367	2.781132
Water/Food	16	Prophet	seasonality = daily, mode = multiplicative	62.935840	6.58161
Water/Food	16	XGBoost	estimators = 700, depth = 6	23.39255	2.00468
Water/Food	16	LSTM		0.6246	0.5562

**Table A9:** Best results of model evaluation on EOD 18-24 raw water per day.

Dataset	Length	Model	Settings	MSE	MAE
Water/Day	35	LLama-3.1-8b	Round6, integers leading 0	63,745.386	96.94633
Water/Day	35	LLama-3-8b	Round6, minimal KE, integer leading 0	64,702	97.65088
Water/Day	35	Falcon-7b	Round7, minimal KE, integer leading 0	61,410.516	108.23203
Water/Day	35	LLama-3.2-1b	Round7, integers leading 0	63,554.199	101.94559
Water/Day	35	LLama-3.2-3b	Round6, pred1	63,255.336	99.65455
Water/Day	35	FEDformer	dm1024 nh8 el4 dl4 df2048 fc3	44,349.059	122.17598
Water/Day	35	Autoformer	dm1024 nh8 el4 dl4 df2048 fc3	71,051.375	195.52776
Water/Day	35	Informer	dm1024 nh8 el4 dl4 df2048 fc3	63,6301.12	752.30859
Water/Day	35	Transformer	dm1024 nh8 el4 dl4 df2048 fc3	635,873.75	752.04865
Water/Day	35	Linear		47,395.52	153.22598
Water/Day	35	NLinear		34,418.758	100.29855
Water/Day	35	DLinear		45,670.336	146.07356
Water/Day	35	PatchTST		30,045.186	88.355118
Water/Day	35	TimeLLM	GPT2	33,996.527	121.36325
Water/Day	35	TimeLLM	LLAMA	27,202.824	103.54283
Water/Day	35	TimeLLM	LLama-3.1-8b	33,246.043	135.12067
Water/Day	35	TimeLLM	LLama-3.2-1b	44,537.82	157.351
Water/Day	35	TimeLLM	LLama-3.2-3b	50,407.285	148.35048
Water/Day	35	TimesFM		69,906.184	155.88891
Water/Day	35	ARIMA	96, 0, 6	317,326.68748	451.2992161
Water/Day	35	Prophet	seasonality = daily, mode = multiplicative	50,732.796193	164,133,299
Water/Day	35	XGBoost	estimators = 700, depth = 6	55,122.3666	120.274808
Water/Day	35	LSTM		226,152.1797	429.03188101

**Table A10:** Best results of model evaluation on EOD 18-24 raw food per day.

Dataset	Length	Model	Settings	MSE	MAE
Food/Day	35	LLama-3.1-8b	Round6	16,931.818	87.94107
Food/Day	35	LLama-3-8b	Round6	16,931.818	87.94107
Food/Day	35	Falcon-7b	Round6	16,931.818	87.94107
Food/Day	35	LLama-3.2-1b	Round6	16,931.818	87.94107
Food/Day	35	LLama-3.2-3b	Round6	16,931.818	87.94107
Food/Day	35	FEDformer	dm1024 nh8 el4 dl4 df2048 fc3	11,468.257	71.640099
Food/Day	35	Autoformer	dm1024 nh8 el4 dl4 df2048 fc3	17,393.186	92.439812
Food/Day	35	Informer	dm1024 nh8 el4 dl4 df2048 fc3	205,548.92	425.98523
Food/Day	35	Transformer	dm1024 nh8 el4 dl4 df2048 fc3	205,643.06	426.08453
Food/Day	35	Linear		11,846.948	75.168358
Food/Day	35	NLinear		6607.9839	48.441002
Food/Day	35	DLinear		11,733.617	74.472069
Food/Day	35	PatchTST		5418.8311	36.753399
Food/Day	35	TimeLLM	GPT2	8365.8789	61.055107
Food/Day	35	TimeLLM	LLAMA	7521.2197	48.208687
Food/Day	35	TimeLLM	LLama-3.1-8b	7828.5215	56.618542
Food/Day	35	TimeLLM	LLama-3.2-1b	12,181.811	79.305267
Food/Day	35	TimeLLM	LLama-3.2-3b	11,142.699	66.228477
Food/Day	35	TimesFM		15,674.906	71.358452
Food/Day	35	ARIMA	96, 0, 6	58,600.825657	194.563275
Food/Day	35	Prophet	seasonality = daily, mode = multiplicative	20,569.6108	124.62285
Food/Day	35	XGBoost	estimators = 700, depth = 6	8432.62189	52.20021
Food/Day	35	LSTM		94,945.739959	280.33244

**Table A11:** Best results of model evaluation on EOD 18-24 raw water per food per day.

Dataset	Length	Model	Settings	MSE	MAE
Water/Food	35	LLama-3.1-8b	Round7, integers leading 0	0.530766	0.403786
Water/Food	35	LLama-3-8b	Round7, minimal KE, integers leading 0	0.543724	0.404424
Water/Food	35	Falcon-7b	Round7	0.342801	0.304580
Water/Food	35	LLama-3.2-1b	Round7, pred1, integers leading 0	0.474231	0.384112
Water/Food	35	LLama-3.2-3b	Round7	0.567289	0.429888
Water/Food	35	FEDformer	dm512 nh8 el4 dl4 df2048 fc2	12.721095	1.6575569
Water/Food	35	Autoformer	dm512 nh8 el4 dl4 df2048 fc2	20.563625	2.1462989
Water/Food	35	Informer	dm512 nh8 el4 dl4 df2048 fc3	0.5531605	0.5554001
Water/Food	35	Transformer	dm512 nh8 el2 dl1 df2048 fc3	0.5594669	0.5674024
Water/Food	35	Linear		6.7625961	1.5873865
Water/Food	35	NLinear		7.9038653	1.5324063
Water/Food	35	DLinear		7.772625	1.8593231
Water/Food	35	PatchTST		8.894248	1.6568558
Water/Food	35	TimeLLM	GPT2	21.213932	2.5848339
Water/Food	35	TimeLLM	LLAMA	14.042688	2.2479351
Water/Food	35	TimeLLM	LLama-3.1-8b	17.969053	2.6226354
Water/Food	35	TimeLLM	LLama-3.2-1b	111.3106	5.1977305
Water/Food	35	TimeLLM	LLama-3.2-3b	42.425476	3.2953618
Water/Food	35	TimesFM		0.5520953	0.4224015
Water/Food	35	ARIMA	96, 0, 6	26.8833622	2.445425
Water/Food	35	Prophet	seasonality = daily, mode = multiplicative	0.212537	0.40059
Water/Food	35	XGBoost	estimators = 700, depth = 6	1.100196	0.396809
Water/Food	35	LSTM		5.0412	1.80235

**Table A12:** Best results of model evaluation on EOD 12-24 on raw water per day.

Dataset	Length	Model	Settings	MSE	MAE
Water/Day	72	LLama-3.1-8b	Round7	127,219.5	179.63584
Water/Day	72	LLama-3-8b	Round6	400,398.2	232.81554
Water/Day	72	Falcon-7b	Round7	99,966.3	188.24978
Water/Day	72	LLama-3.2-1b	Round6	215,273.1	211.50824
Water/Day	72	LLama-3.2-3b	Round6	128,601.8	183.02409
Water/Day	72	FEDformer	dm1024 nh8 el4 dl4 df2048 fc3	67,055.773	150.94092
Water/Day	72	Autoformer	dm512 nh8 el4 dl4 df2048 fc2	70,955.727	180.86993
Water/Day	72	Informer	dm1024 nh8 el4 dl4 df2048 fc3	456,403.38	610.25104
Water/Day	72	Transformer	dm1024 nh8 el4 dl4 df2048 fc3	455,644.03	609.69543
Water/Day	72	Linear		99,236.414	236.90807
Water/Day	72	NLinear		39,264.383	117.4375
Water/Day	72	DLinear		67,357.328	177.82516
Water/Day	72	PatchTST		30,868.742	99.920082
Water/Day	72	TimeLLM	GPT2	36,441.277	115.92726
Water/Day	72	TimeLLM	LLAMA	48,275.848	135.40031
Water/Day	72	TimeLLM	LLama-3.1-8b	69,104.68	189.53014
Water/Day	72	TimeLLM	LLama-3.2-1b	62,497.641	170.28175
Water/Day	72	TimeLLM	LLama-3.2-3b	41,100.262	129.60506
Water/Day	72	TimesFM		97,844.638	192.79371
Water/Day	72	ARIMA	96, 0, 6	316,569.758632	450.2248
Water/Day	72	Prophet	seasonality = daily, mode = multiplicative	32,817.351578	131.943
Water/Day	72	XGBoost	estimators = 700, depth = 6	126,232.35895	164.597509
Water/Day	72	LSTM		158,568.514725	329.969951

**Table A13:** Best results of model evaluation on EOD 12-24 on raw food per day.

Dataset	Length	Model	Settings	MSE	MAE
Food/Day	72	LLama-3.1-8b	Round6	43,614.63	168.08182
Food/Day	72	LLama-3-8b	Round6	43,614.63	168.08182
Food/Day	72	Falcon-7b	Round6	43,614.63	168.08182
Food/Day	72	LLama-3.2-1b	Round6	43,614.63	168.08182
Food/Day	72	LLama-3.2-3b	Round6	43,614.63	168.08182
Food/Day	72	FEDformer	dm1024 nh8 el4 dl4 df2048 fc3	15,813.656	96.876381
Food/Day	72	Autoformer	dm512 nh8 el2 dl1 df2048 fc3	16,074.986	95.071198
Food/Day	72	Informer	dm512 nh8 el4 dl4 df2048 fc2	11,043.87	71.900696
Food/Day	72	Transformer	dm1024 nh8 el4 dl4 df2048 fc3	8197.9727	60.516228
Food/Day	72	Linear		32,399.889	142.77518
Food/Day	72	NLinear		13,294.714	70.150322
Food/Day	72	DLinear		16,815.316	94.899933
Food/Day	72	PatchTST		8156.7891	49.072395
Food/Day	72	TimeLLM	GPT2	14,227.891	81.751991
Food/Day	72	TimeLLM	LLAMA	15,507.049	88.383728
Food/Day	72	TimeLLM	LLama-3.1-8b	10,399.145	68.121513
Food/Day	72	TimeLLM	LLama-3.2-1b	10,741.901	67.632515
Food/Day	72	TimeLLM	LLama-3.2-3b	11,974.566	76.812675
Food/Day	72	TimesFM		26,058.219	107.60436
Food/Day	72	ARIMA	96, 0, 6	58,525.7958	194.235154
Food/Day	72	Prophet	seasonality = daily, mode = multiplicative	13,867.42374	99.8531971
Food/Day	72	XGBoost	estimators = 700, depth = 6	10,311.58899	61.2380111
Food/Day	72	LSTM		61,487.02896	208.947407

**Table A14:** Best results of model evaluation on EOD 12-24 on raw water per food.

Dataset	Length	Model	Settings	MSE	MAE
Water/Food	72	LLama-3.1-8b	Round6	29.75216	1.868294
Water/Food	72	LLama-3-8b	Round6	30.96716	1.946766
Water/Food	72	Falcon-7b	Round6	26.77479	1.512218
Water/Food	72	LLama-3.2-1b	Round7	31.18094	1.781682
Water/Food	72	LLama-3.2-3b	Round6	31.00623	1.874792
Water/Food	72	FEDformer	dm512 nh8 el2 dl1 df2048 fc3	47.567806	3.6072099
Water/Food	72	Autoformer	dm512 nh8 el4 dl4 df2048 fc2	47.90118	3.6683018
Water/Food	72	Informer	dm1024 nh8 el4 dl4 df2048 fc3	4.2334499	0.7910022
Water/Food	72	Transformer	dm512 nh8 el2 dl1 df2048 fc3	4.6746197	0.9696611
Water/Food	72	Linear		18.464836	2.503298
Water/Food	72	NLinear		31.623196	2.3313577
Water/Food	72	DLinear		17.509649	2.6272669
Water/Food	72	PatchTST		19.467442	2.3075855
Water/Food	72	TimeLLM	GPT2	128.50887	5.5041637
Water/Food	72	TimeLLM	LLAMA	26.97904	2.9874995
Water/Food	72	TimeLLM	LLama-3.1-8b	49.222603	3.8950772
Water/Food	72	TimeLLM	LLama-3.2-1b	71.824181	4.582509
Water/Food	72	TimeLLM	LLama-3.2-3b	110.70022	5.2826319
Water/Food	72	TimesFM		1.9870864	0.8731274
Water/Food	72	ARIMA	96, 0, 6	27.13487	2.46185
Water/Food	72	Prophet	seasonality = daily, mode = multiplicative	0.23685	0.40524
Water/Food	72	XGBoost	estimators = 700, depth = 6	9.53428099	0.7338821
Water/Food	72	LSTM		16.30125233	2.889976

**Table A15:** Best results of model evaluation on EOD 08-24 on raw water per day.

Dataset	Length	Model	Settings	MSE	MAE
Water/Day	94	LLama-3.1-8b	Round6	134,045.0	229.66190
Water/Day	94	LLama-3-8b	Round6, minimal KE	132,584.4	228.46576
Water/Day	94	Falcon-7b	Round6	160,179.5	281.47873
Water/Day	94	LLama-3.2-1b	Round6	181,587.5	268.71460
Water/Day	94	LLama-3.2-3b	Round7	136,423.6	237.23671
Water/Day	94	FEDformer	dm1024 nh8 el4 dl4 df2048 fc3	154,820.84	205.27248
Water/Day	94	Autoformer	dm1024 nh8 el4 dl4 df2048 fc3	106,292.59	231.01073
Water/Day	94	Informer	dm1024 nh8 el4 dl4 df2048 fc3	375,410.12	538.77234
Water/Day	94	Transformer	dm1024 nh8 el4 dl4 df2048 fc3	375,288.69	538.6684
Water/Day	94	Linear		151,499.34	290.81503
Water/Day	94	NLinear		81,248.977	191.62004
Water/Day	94	DLinear		81,626.609	196.50258
Water/Day	94	PatchTST		56,002.957	136.36386
Water/Day	94	TimeLLM	GPT2	62,078.516	158.99992
Water/Day	94	TimeLLM	LLAMA	64,618.055	171.73296
Water/Day	94	TimeLLM	LLama-3.2-1b	68,709.617	178.30528
Water/Day	94	TimeLLM	LLama-3.2-3b	69,128.562	180.73517
Water/Day	94	TimesFM		167,282.64	308.11604
Water/Day	94	ARIMA	96, 0, 6	314,858.7631	448.9585
Water/Day	94	Prophet	seasonality = daily, mode = multiplicative	23,627.412932	107.2348
Water/Day	94	XGBoost	estimators = 700, depth = 6	37,955.39699	108.960082
Water/Day	94	LSTM		54,214.290572	193.1003371

**Table A16:** Best results of model evaluation on EOD 08-24 on raw food per day.

Dataset	Length	Model	Settings	MSE	MAE
Food/Day	94	LLama-3.1-8b	Round6	106,993.9	273.90292
Food/Day	94	LLama-3-8b	Round6	106,993.9	273.90292
Food/Day	94	Falcon-7b	Round6	106,993.9	273.90292
Food/Day	94	LLama-3.2-1b	Round6	106,993.9	273.90292
Food/Day	94	LLama-3.2-3b	Round6	106,993.9	273.90292
Food/Day	94	FEDformer	dm1024 nh8 el4 dl4 df2048 fc3	63,938.793	203.65533
Food/Day	94	Autoformer	dm512 nh8 el4 dl4 df2048 fc2	34,084.82	144.66563
Food/Day	94	Informer	dm1024 nh8 el4 dl4 df2048 fc3	112,796.54	289.76105
Food/Day	94	Transformer	dm1024 nh8 el4 dl4 df2048 fc3	112,717.09	289.63867
Food/Day	94	Linear		55,614.445	187.25125
Food/Day	94	NLinear		54,986.531	183.73718
Food/Day	94	DLinear		24,125.869	119.84232
Food/Day	94	PatchTST		9165.3945	64.747391
Food/Day	94	TimeLLM	GPT2	11,608.899	75.451408
Food/Day	94	TimeLLM	LLAMA	10,372.419	66.009544
Food/Day	94	TimeLLM	LLama-3.2-1b	18,642.949	98.507324
Food/Day	94	TimeLLM	LLama-3.2-3b	10,981.992	69.961159
Food/Day	94	TimesFM		108,090.62	275.52387
Food/Day	94	ARIMA	96, 0, 6	58,241.02083	193.74541
Food/Day	94	Prophet	seasonality = daily, mode = multiplicative	10,607.051844	83.265704
Food/Day	94	XGBoost	estimators = 700, depth = 6	6665.1874871	52.4635754
Food/Day	94	LSTM		18,821.261799	119.3361072

**Table A17:** Best results of model evaluation on EOD 08-24 on raw water per food.

Dataset	Length	Model	Settings	MSE	MAE
Water/Food	94	LLama-3.1-8b	Round6	2064.6154	25.13210
Water/Food	94	LLama-3-8b	Round6	2407.4668	26.75854
Water/Food	94	Falcon-7b	Round6	1017.1714	18.91526
Water/Food	94	LLama-3.2-1b	Round7	1668.2446	23.90957
Water/Food	94	LLama-3.2-3b	Round7, integers lead 0	18.56463	2.491583
Water/Food	94	FEDformer	dm512 nh8 el2 dl1 df2048 fc3	107.50979	5.9102168
Water/Food	94	Autoformer	dm512 nh8 el2 dl1 df2048 fc3	103.11153	5.7164969
Water/Food	94	Informer	dm512 nh8 el4 dl4 df2048 fc3	9.4249907	1.1121691
Water/Food	94	Transformer	dm1024 nh8 el4 dl4 df2048 fc3	9.66008	0.9591334
Water/Food	94	Linear		32.426571	3.2802207
Water/Food	94	NLinear		532.24011	12.311095
Water/Food	94	DLinear		33.396038	3.2424126
Water/Food	94	PatchTST		48.766281	3.8374641
Water/Food	94	TimeLLM	GPT2	54.385811	4.5503159
Water/Food	94	TimeLLM	LLAMA	65.39135	4.5003433
Water/Food	94	TimeLLM	LLama-3.2-1b	154.21689	6.2390552
Water/Food	94	TimeLLM	LLama-3.2-3b	56.128967	4.3136649
Water/Food	94	TimesFM		362.32859	9.1224683
Water/Food	94	ARIMA	96, 0, 6	2,727,683,668	2.46731
Water/Food	94	Prophet	seasonality = daily, mode = multiplicative	15.434231	0.858939
Water/Food	94	XGBoost	estimators = 700, depth = 6	95.4685184	4.0301748
Water/Food	94	LSTM		103.346606	5.0909842

This test forecasts the values between 06:00:00 and 08:00:00 using the start of the day value as input. This time is critical, as it is when the turkeys are just waking up.

**Table A18:** Best results of model evaluation on EOD 06-08 on raw water per day.

Dataset	Length	Model	Settings	MSE	MAE
Water/Day	12	LLama-3.1-8b	Round6	4250.3726	38.61718
Water/Day	12	LLama-3-8b	Round6, minimal KE	4225.8384	38.21875
Water/Day	12	Falcon-7b	Round6, minimal KE	4263.4897	38.72395
Water/Day	12	LLama-3.2-1b	Round7	4262.8374	38.78515
Water/Day	12	LLama-3.2-3b	Round7	4235.9282	38.51432
Water/Day	12	FEDformer	dm512 nh8 el2 dl1 df2048 fc3	4264.6294	46.152172
Water/Day	12	Autoformer	dm1024 nh8 el4 dl4 df2048 fc3	4363.5503	45.404327
Water/Day	12	Informer	dm1024 nh8 el4 dl4 df2048 fc3	43,233.488	177.9469
Water/Day	12	Transformer	dm1024 nh8 el4 dl4 df2048 fc3	43,026.168	177.40883
Water/Day	12	Linear		14,518.612	92.228477
Water/Day	12	NLinear		7100.5547	57.105877
Water/Day	12	DLinear		11,145.277	76.910149
Water/Day	12	PatchTST		2217.4202	23.792437
Water/Day	12	TimeLLM	GPT2	4171.312	44.646255
Water/Day	12	TimeLLM	LLAMA	22,659.805	115.91663
Water/Day	12	TimeLLM	LLama-3.1-8b	6940.5845	62.111256
Water/Day	12	TimeLLM	LLama-3.2-1b	9221.1641	72.825569
Water/Day	12	TimeLLM	LLama-3.2-3b	33,299.645	127.93154
Water/Day	12	TimesFM		31,208.614	113.44911
Water/Day	12	ARIMA	96, 0, 6	314,669.637	448.9622
Water/Day	12	Prophet	seasonality = daily, mode = multiplicative	6706.82274	64.3016
Water/Day	12	XGBoost	estimators = 700, depth = 6	36,346.71904	68.90266777
Water/Day	12	LSTM		34,974.1794334	173.6668

**Table A19:** Best results of model evaluation on EOD 06-08 on raw food per day.

Dataset	Length	Model	Settings	MSE	MAE
Food/Day	12	LLama-3.1-8b	Round6	606.57551	8.601563
Food/Day	12	LLama-3-8b	Round6	606.57551	8.601563
Food/Day	12	Falcon-7b	Round6	606.57551	8.601563
Food/Day	12	LLama-3.2-1b	Round6	606.57551	8.601563
Food/Day	12	LLama-3.2-3b	Round6	606.57551	8.601563
Food/Day	12	FEDformer	dm1024 nh8 el4 dl4 df2048 fc3	3667.6523	53.018097
Food/Day	12	Autoformer	dm1024 nh8 el4 dl4 df2048 fc3	1733.0547	30.34667
Food/Day	12	Informer	dm1024 nh8 el4 dl4 df2048 fc3	2889.9724	27.680847
Food/Day	12	Transformer	dm512 nh8 el4 dl4 df2048 fc2	3113.7939	29.272665
Food/Day	12	Linear		2254.8643	36.60244
Food/Day	12	NLinear		1876.588	32.061527
Food/Day	12	DLinear		2043.4894	33.071217
Food/Day	12	PatchTST		744.31055	17.705568
Food/Day	12	TimeLLM	GPT2	2182.7136	36.632145
Food/Day	12	TimeLLM	LLAMA	829.46259	19.06687
Food/Day	12	TimeLLM	LLama-3.1-8b	1670.5341	29.97143
Food/Day	12	TimeLLM	LLama-3.2-1b	849.55096	22.064957
Food/Day	12	TimeLLM	LLama-3.2-3b	926.60138	22.062468
Food/Day	12	TimesFM		63,453.254	186.62499
Food/Day	12	ARIMA	96, 0, 6	58,720.45933	193.85355
Food/Day	12	Prophet	seasonality = daily, mode = multiplicative	718.2456	20.33473
Food/Day	12	XGBoost	estimators = 700, depth = 6	950.585651	21.6275
Food/Day	12	LSTM		21,673.792321	141.724656

**Table A20:** Best results of model evaluation on EOD 06-08 on raw water per food.

Dataset	Length	Model	Settings	MSE	MAE
Water/Food	12	LLama-3.1-8b	Round6	65.23934	3.512001
Water/Food	12	LLama-3-8b	Round6	65.56091	3.466901
Water/Food	12	Falcon-7b	Round7	64.68107	3.4630734
Water/Food	12	LLama-3.2-1b	Round7	65.25236	3.502396
Water/Food	12	LLama-3.2-3b	Round6	65.26582	3.495886
Water/Food	12	FEDformer	dm512 nh8 el4 dl4 df2048 fc3	76.843819	4.4923301
Water/Food	12	Autoformer	dm512 nh8 el2 dl1 df2048 fc3	74.666283	4.2812572
Water/Food	12	Informer	dm1024 nh8 el4 dl4 df2048 fc3	211.39128	7.1620622
Water/Food	12	Transformer	dm1024 nh8 el4 dl4 df2048 fc3	192.72319	6.7343025
Water/Food	12	Linear		313.64319	9.3907223
Water/Food	12	NLinear		95.085266	4.3722696
Water/Food	12	DLinear		325.85709	9.6598787
Water/Food	12	PatchTST		141.24937	4.6548424
Water/Food	12	TimeLLM	GPT2	217.675	7.4429684
Water/Food	12	TimeLLM	LLAMA	165.22368	6.4503074
Water/Food	12	TimeLLM	LLama-3.1-8b	172.60667	5.9101272
Water/Food	12	TimeLLM	LLama-3.2-1b	147.49525	5.9639492
Water/Food	12	TimeLLM	LLama-3.2-3b	169.36555	5.6974831
Water/Food	12	TimesFM		75.693913	4.0768743
Water/Food	12	ARIMA	96, 0, 6	27.3321	2.46678
Water/Food	12	Prophet	dseasonality = daily, mode = multiplicative	66.66029	6.018013
Water/Food	12	XGBoost	estimators = 700, depth = 6	104.99004	4.794791
Water/Food	12	LSTM		388.5438684	10.96561
